# Effects of Processing on Polyphenolic and Volatile Composition and Fruit Quality of Clery Strawberries

**DOI:** 10.3390/antiox9070632

**Published:** 2020-07-17

**Authors:** Stefania Garzoli, Francesco Cairone, Simone Carradori, Andrei Mocan, Luigi Menghini, Patrizia Paolicelli, Gunes Ak, Gokhan Zengin, Stefania Cesa

**Affiliations:** 1 Department of Drug Chemistry and Technologies, University “Sapienza” of Rome, P.le Aldo Moro 5, 00185 Rome, Italy; stefania.garzoli@uniroma1.it (S.G.); francesco.cairone@uniroma1.it (F.C.); patrizia.paolicelli@uniroma1.it (P.P.); 2 Department of Pharmacy, “G. d’Annunzio” University of Chieti-Pescara, Via dei Vestini 31, 66100 Chieti, Italy; luigi.menghini@unich.it; 3 Faculty of Pharmacy, “Iuliu Hațieganu” University of Medicine and Pharmacy, 8 Victor Babeș Street, 400012 Cluj-Napoca, Romania; Mocan.Andrei@umfcluj.ro; 4 Department of Biology, Science Faculty, Selcuk University, Konya 42130, Turkey; akguneselcuk@gmail.com (G.A.); gokhanzengin@selcuk.edu.tr (G.Z.)

**Keywords:** Clery strawberry, food processing, Polyphenols, multi-methodological evaluation, HS-GC/MS analysis, PCA

## Abstract

Strawberries belonging to cultivar Clery (*Fragaria* *x* *ananassa* (Duchesne ex Weston)), cultivated in central Italy were subjected to a multi-methodological experimental study. Fresh and defrosted strawberries were exposed to different processing methods, such as homogenization, thermal and microwave treatments. The homogenate samples were submitted to CIEL*a*b* color analysis and Head-Space GC/MS analysis to determine the impact of these procedures on phytochemical composition. Furthermore, the corresponding strawberry hydroalcoholic extracts were further analyzed by HPLC-DAD for secondary metabolites quantification and by means of spectrophotometric in vitro assays to evaluate their total phenolic and total flavonoid contents and antioxidant activity. These chemical investigations confirmed the richness in bioactive metabolites supporting the extraordinary healthy potential of this fruit as a food ingredient, as well as functional food, highlighting the strong influence of the processing steps which could negatively impact on the polyphenol composition. Despite a more brilliant red color and aroma preservation, non-pasteurized samples were characterized by a lower content of polyphenols and antioxidant activity with respect to pasteurized samples, as also suggested by the PCA analysis of the collected data.

## 1. Introduction

The healthy potential associated with the daily consumption of vegetables and fruits has attracted increasing interest in the last two decades [1,2]. Particularly, berries are appreciated for their healthy potential in relation to their high content of polyphenols, such as cinnamic acids and flavonoids, between which contain, of particular interest, anthocyanins. Strawberries display a rich content in nutritive and non–nutritive bioactive components, correlated to antioxidant potential and disease prevention along with excellent characters of flavor, color and taste. All these aspects make them one of the preferred available fruits, with an annual production of about five million tons per year worldwide [3]. Many recent studies underlined the healthy potential of strawberries [4,5,6]. A dietetic regime in which strawberry derivatives were plenty and regularly introduced could prevent inflammation onset and promote the reduction of obesity–related disorders and even cardiovascular diseases and neurodegeneration [7].

Many different strawberry cultivars are available on the market in different countries, each with their own peculiarities, and there are many variables which could influence the final content of specific molecules and their health potential, such as humidity and pedoclimatic conditions, fertilizers and other growing parameters [5]. Moreover, these fruits are not always consumed fresh, especially in relation to their limited seasonal availability. In this view, a dominant role is played by the applied procedures to obtain processed products, such as juices and jams, whose impact on the labile components of phytocomplex must be strictly controlled in order to prevent degradation and fragmentation that can occur, for example, to phenolic compounds at high temperatures.

Among the 73 strawberry cultivars available in Italy (Gazzetta Ufficiale della Repubblica Italiana n. 217, 2017), and many other available worldwide, Clery strawberries, cultivated in central Italy, were chosen as the study material. Clery strawberry is a recent variety (1998) obtained by breeding “Sweet Charlie” and “Marmolada” cultivars and has been authorized for commercial use since 2002. Its main characters are its regular shape, sweetness and taste, largely appreciated by its consumers, coupled with its resistance to cold and adverse climate conditions [8]. This strawberry variety was previously the object of other research studies. Two studies showed the presence of polyphenolic compounds, among which catechins and procyanidins, quercetin and kaempferol glucuronides, ellagitannins, pelargonidin–3–glucoside and pelargonidin–3–rutinoside accounted for the most represented molecules [9,10].

Nowicka et al. [11], who recently analyzed fruits selected from 90 strawberry cultivars, concluded that ellagitannins and procyanidins deeply influence the antioxidant capacity of strawberries evaluated by the ABTS test. In the review by Afrin et al. [5], the highest reported components are represented by proanthocyanidins (up to 163 mg/100 g fresh weight [fw]), ellagitannins (up to 83 mg/100 g fw) and anthocyanins (up to 66 mg/100 g fw). The authors, who evaluated the health benefits, focusing on the existing clinical studies on cardiovascular disease, metabolic syndrome, obesity and diabetes, neuroprotection and antimicrobial activity, besides antioxidant and anti-inflammatory activity, also consider strawberries a rich source of phenolic acids (up to 13 mg/100 g fw). Finally, kaempferol and quercetin, whose bioactivity as neuroprotective agents and as enhancers of adiponectin secretion by PPARγ, was also largely documented [12,13], accounting for up to 3 and 5 mg/100 g fw, respectively. As reported by da Silva et al. [14], anthocyanin pigments were mainly represented by pelargonidin and cyanidin aglycones, mostly glycosylated by glucose and rutinose and less frequently by arabinose and rhamnose. Only rarely were some acylated anthocyanins also detected; therefore, despite a very high content of anthocyanins, the represented composition is quite simple. These latter components, besides the healthy properties, account for the brilliant and consumer-appealing red color, whereas other extraordinary flavoring compounds characterize the strawberry aroma, both representing a delicate issue in case of the applied processing.

The Clery fruit quality was evaluated in terms of ripening, pre–harvest and post–harvest conditions [15,16], and the impact of different genotypes, fertilizers and harvesting date on fruit yield, sugars, phenolic compounds and anthocyanins content was evaluated. As stated by Tomić et al. [15], if genotype–cultivar represents the determinant factor in fruit quality, ripening, pre– and post–harvest treatments, processing and storage deeply influence the phytocomplex modification and the change–related consequences; therefore, the aim of this work was to evaluate in which way processing modifies the strawberry quality. In this context, diverse processing steps were applied and differently combined. Some interesting quality parameters, significant both for the consumer choice and for the healthy strawberry potential, such as total polyphenol, flavonoid and phenolic acid content, anthocyanin content, flavor and color were chosen to be assayed by using different analytical techniques.

Starting with the increasing interest devoted in the last twenty years to the healthy properties generally recognized to polyphenols and conscious of their limiting factors due to bioavailability and human metabolism, our aim was to evaluate how and how deeply processing affects the starting food quality. The effects of similar processing methods were previously evaluated in blueberries and goji berries [17,18]. In the present work, the effects of different homogenization, thermal and microwave treatments applied on Clery strawberries of Italian origin were monitored in terms of color and aroma changes and as variations of the polyphenolic content and antioxidant activity.

## 2. Materials and Methods

### 2.1. Materials

Commercial samples of strawberry fruits, *Fragaria x ananassa* (Duchesne ex Weston) and Duchesne ex Rozier (syn *Fragaria x ananassa* Duchesne) of the patent cultivar “Clery”, were purchased at the farm “Fragole di Carchitti” a local company that controls all the productive chain, from cultivation to sale (Palestrina, RM, 41,83274 °N, 12.88178 °E). Plant material was harvested as single crop on 28 May, 2019, macroscopically selected as ready for food consumption, which reflects the full ripening status, and immediately stored at 4–6 °C. The day after the harvest, the strawberries fruits started to be used as fresh material (series f, fresh) and treatments were completed in a week. After gentle cleaning to remove impurities and green parts, strawberries were carefully washed with tap water and dried on paper. All other material was immediately frozen at −80 °C and then stored at −18 °C and used for test just after the defreezing (series d) within two months from the harvest date. Bidistilled water, ethanol, 85% formic acid, acetonitrile RS and reference compounds for HPLC were purchased from Merck life Science s.r.l. (Milan, Italy).

### 2.2. Processing

**Figure 1:**
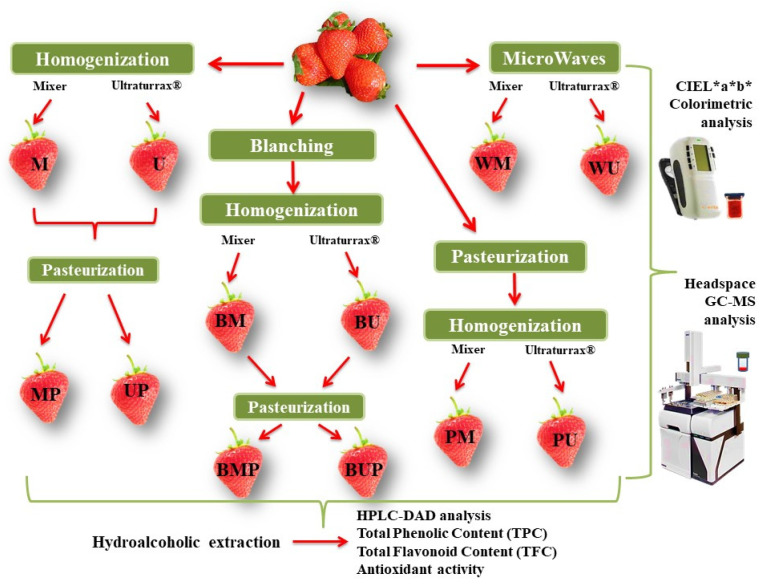
Flow chart of the applied treatments and the performed analyses on fully ripe fresh or defrosted Clery strawberries.

The fresh or thawed out fruits were processed and extracted according to the synoptic workflow represented in Figure 1. For each homogenization process, an aliquot of approximately 25 g was used. Coming from the same farm and the same crop, picked as fully ripe and characterized by similar size (each about 7–10 g), the strawberries were considered a homogeneous sample and were weighed as whole fruits in order to prepare the aliquots.

The blanching process consists in steamed plant material for 3 min at 85 °C, cooled at room temperature and then homogenized for 2 min either by a domestic mixer at 16,000 rpm (BM) or by a T18 Ultraturrax^®^ homogenizer (IKA^®^, Staufen, Germany) at 10,000 rpm (BU) and subsequently submitted to a pasteurization treatment at 85 °C for 3 min (BMP, BUP). A parallel process starts with homogenization of plant material for 2 min (using domestic mixer or Ultraturrax^®^ homogenizer, samples M and U, respectively) and the resulting purees heated at 85 °C for 3 min (samples MP and UP, respectively). The third approach starts with pasteurization followed by homogenization (as above described, samples PM and PU, respectively). The last experimental condition starts with pre-heating treatment in a domestic microwave oven (Samsung, T181) at 450 W for 1 min (final reached T by food was 70 ± 2 °C) and then samples were homogenized (as above described, samples WM and WU, respectively). All the treatments were performed in parallel on fresh and defrosted fruits. After each type of treatment, an aliquot of the purees was used for the colorimetric CIEL*a*b* and for the head space-gas chromatography/mass spectrometry (HS–GC/MS) analyses.

### 2.3. pH Measurement

Aliquots of about 1 g of the purees were transferred in conical tubes and centrifuged (5000g for 10 min). The pH of the supernatant was determined using a Metrohm E632 pH–meter (Metrohm Italiana S.r.l., Rome, Italy).

### 2.4. Colorimetric Analysis

The homogenates were analyzed for their color character, with a colorimeter X–Rite (SP–62, GmbH, Regensdorf, Switzerland), equipped with D65 illuminant and an observer angle of 10°. Cylindrical coordinates C*_ab_ and h_ab_ are calculated from a* and b* as previously indicated [18].

### 2.5. HS–GC/MS Analyses

Gas chromatographic/mass spectrometric (GC/MS) analysis was carried out using a Turbomass Clarus 500 GC–MS/GC-FID from Perkin Elmer instruments (Waltham, MA, USA). A Stabilwax fused–silica capillary column (Restek, Bellefonte, PA, USA) (60 m × 0.25 mm, 0.25 um film thickness) was used with helium as carrier gas (1.0 mL/min). GC oven temperature was kept at 40 °C and programmed to 220 °C at a rate of 5 °C/min, and kept constant at 220 °C for 5 min. All mass spectra were recorded in the electron impact ionization (EI) at 70 eV. Mass range was from 30 to 400 *m*/*z*. To examine the volatile fraction of strawberry samples, a Perkin–Elmer Headspace (HS) Turbomatrix 40 autosampler connected to a Clarus 500 GC-MS was used for the headspace analysis. This technique is ideal for sampling volatiles present in solid and liquid matrices [19,20].

The sampling procedure was thus carried out: sliced fruits and homogenates were collected into a 20 mL vial and tightly sealed immediately with crimp aluminum caps and 20 mm white rubber septa (Supelco, USA) using a vial crimper. With the intent to optimize the headspace procedure for the determination of a broader number of volatile organic compounds (VOCs) from strawberry samples, some parameters were adjusted as follows: thermostating temperature was 60 °C; thermostating time was 20 min; the pressurization time was 3.0 min; needle temperature was 90 °C; and the injection time was 0.3 min.

For the identification of the volatile fraction of strawberry samples, a Perkin-Elmer Headspace (HS) Turbomatrix 40 autosampler connected to a Clarus 500 GC–MS was used for the headspace analysis. This technique is ideal for sampling volatiles present in solid and liquid matrices [19,20]. With the intent to optimize the headspace sampling procedure for the determination of volatile organic compounds (VOCs), some parameters, such as equilibration time, temperature and head space analysis duration, were adjusted [21]. The sampling procedure was thus carried out: sliced fruits and homogenates were collected into a 20 mL vial and tightly sealed immediately with crimp aluminum caps and 20-mm white rubber septa (Supelco, PA, USA) using a vial crimper. The samples were incubated at 60 °C for 20 min prior the injection. The identification of the volatile main components was obtained by comparison of their linear retention indices (LRIs) and spectral mass with those reported in digital libraries data (Wiley 02 and Nist) of the GC/MS system. The LRI of each compound was calculated using a mixture of aliphatic hydrocarbons (C_8_–C_30_, Ultrasci) injected directly into GC injector under the same conditions described above. Relative percentages of the separated constituents were calculated from integration of the peak areas in the GC chromatograms without the use of an internal standard or correction factors using the same instrumentation with the FID detector configuration. GC/MS analysis of each strawberry sample was carried out twice.

### 2.6. Extraction of Polyphenols

According to Cesa et al. [17] with some modifications, an aliquot (5 g) of each sample was extracted for 1 h, at room temperature and in the dark, under stirring with a hydroalcoholic acidified mixture (15 mL of ethanol/5% formic acid in water, in the ratio 70:30, *v*/*v*). Then, the suspension was filtered, and the residue was washed. The separated solution was slightly concentrated under reduced pressure at 40 °C with a rotary evaporator. Filtered extract was adjusted to a final volume of 20 mL with the same solvent and directly analyzed by HPLC–DAD.

### 2.7. HPLC-DAD Analysis

HPLC analysis was carried out by a Perkin–Elmer (Waltham, MA, USA) apparatus equipped with a series LC 200 pump, a series 200 diode array detector and a series 200 autosampler. Data acquisition and processing were carried out with a Perkin–Elmer Totalchrom software. The chromatographic separation was performed as previously described [22]. In brief, a Luna RP18 column (250 × 4.6 mm, i.d. 5 μm) and a mobile phase, consisting of acetonitrile (A) and acidic water solution (B) in gradient, were used. The detection wavelengths were set at 520 nm for the detection of anthocyanins, at 360 nm for the detection of other flavonoids and at 280 nm for phenolic acids, catechins and other polyphenolic components. The injection volume for each extract was 10 μL. Pelargonidin-3–glucoside, identified at 520 nm in each sample, was quantified by an external-matrix matched calibration method on the basis of the area ratios respect to the pure chemical standard (R^2^ = 0.9984). The other anthocyanins were calculated as the sum of all the chromatographic peaks identified at 520 nm. Calibration curves were built and used for quantitation of polyphenols, using, at 280 nm, epicatechin (R^2^ = 0.9878), caffeic acid (R^2^ = 0.9984), *p*–coumaric acid (R^2^ = 0.9879) and ferulic acid (R^2^ = 0.9974) and, at 360 nm, rutin (R^2^ = 0.9986) and quercetin-3-D-galactoside (R^2^ = 0.9999) as reference standards.

### 2.8. Total Phenolic Content (TPC), Total Flavonoid Content (TFC) and Antioxidant Assays

To obtain the total amount of these polyphenolic groups in the samples, colorimetric assays were used as described in our previous paper [23]. Gallic acid (GAE, Sigma–Aldrich, Germany) and rutin (RE, Sigma–Aldrich, Germany) were used as standards for phenols and flavonoids, respectively. To detect antioxidant and metal chelating properties, we used several chemical assays including different mechanisms, namely, radical scavenging, reducing power and metal chelating. Trolox (TE) and ethylenediaminetetraacetic acid (EDTA) were used as standard antioxidant compounds and experimental results are expressed as activity unit equivalents of these compounds [23].

### 2.9. Statistical Analysis

All analyses were carried out in quadruplicate. The data collected are presented as average values and completed by the standard deviations. One-way analysis of variance (ANOVA), followed by Tukey’s post-hoc test, was employed to assess significant differences (*p* < 0.05) using GraphPad Prism version 5.01 for Windows (GraphPad Software, San Diego, CA, USA). The principal component analysis (PCA) was performed with XLSTAT Version 2020 software.

## 3. Results and Discussion

### 3.1. Processing and Aim of the Work

Two different grinding procedures followed or preceded by two different thermal treatments of pasteurization and steam blanching, or preceded by a microwave treatment, were applied to the selected fruits. A part of the selected fruits was processed immediately after the harvesting. Otherwise, these were frozen at −80 °C and stored at −20 °C, until the thawing and the immediate processing were performed. All the adopted treatments, freezing and thawing, grinding with a domestic mixer or with a highspeed industrial homogenizer, pasteurization and blanching performed at 85° C for 3 min and the microwave treatment performed at 450 W for 1 min represented some classical food processing procedures. Berries, such as strawberries, are often subjected to these treatments during the preparation of jellies or juices in order to reduce the bacterial load, as well as to prevent spontaneous and enzymatic oxidation that could affect organoleptic characters and other quality parameters. Even if the intent is to protect foodstuff, all the adopted steps could exert an influence on the polyphenolic and aromatic content. The aim of the present work was to compare the effects of different work processes and sequences on qualitative and quantitative composition and to define the optimal method to preserve the original color and aroma, which deeply influence the consumer’s choice and the healthy peculiarities.

Previous works, performed on blueberries [17] and goji berries [18], indicated that significant differences could be found in terms of color between differently ground food samples and that more marked differences could be highlighted if the homogenization procedures are combined with different thermal procedures. Here, moreover, the microwave treatment was introduced and the thermal treatments were differently combined and the effects of freezing and thawing were also tested.

The two adopted analytic methods, CIEL*a*b* and HS-GC-MS, allowed us to analyze directly the fruit homogenates returning a sample photograph prior to any impact due to the extraction procedure. Moreover, the HPLC-DAD analysis of the hydroalcoholic extracts obtained by the homogenates, allowed us to complete and compare the data by the colorimetric analysis, giving information about any compositional change of the polyphenolic components. A correlation was finally attempted to be made among all the obtained results by the different used analytical procedures and the differently combined applied procedures.

### 3.2. pH Measurements

The pH of all the obtained homogenates was measured, to verify if differences among differently treated samples could justify consequences in terms of color change, being anthocyanins color expression deeply influenced by pH variation. All the measured pH ranged between 3.7 and 4.0, showing that the detected color changes cannot be attributed to pH variations.

### 3.3. Color Analysis

Color expressed by a foodstuff, besides giving information about its peculiar origin and the nature of its pigment content, could be deeply influenced by the processing. As is well known, fruits including strawberries, which are only available in a very limited seasonal period, are subjected, among many other berries, to significant work–up with the aim to obtain jellies, juices or other storable products. The result of these transformations impacts the organoleptic characters of the final foodstuff which, besides the great influence played on the consumer’s choice, deeply modify the chemical composition.

**Figure 2:**
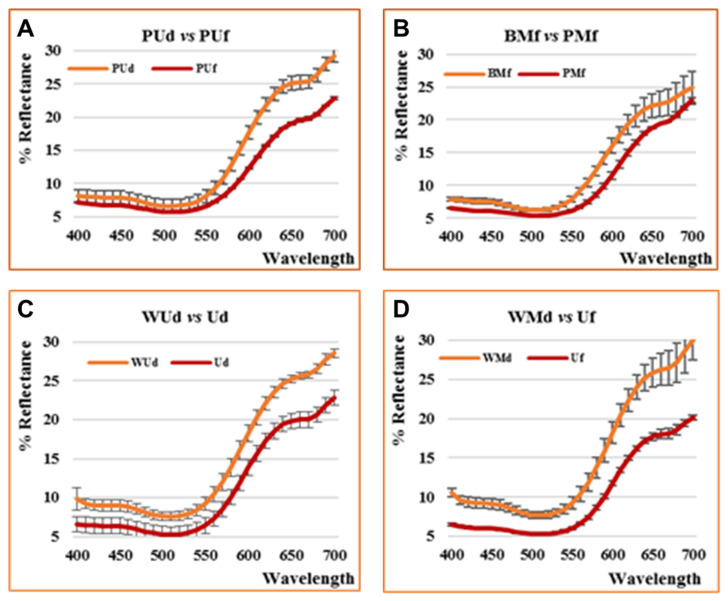
Reflectance curves of some selected homogenates: (**A**) comparison between PUd and PUf; (**B**) comparison between BMf and PMf; (**C**) comparison between WUd and Ud; (**D**) comparison between WMd and Uf. Each reported profile represents the mean of four measurements.

The main pigments of strawberry phytocomplex are represented by anthocyanins and polyphenols, which also represent the most important molecules in terms of healthy properties. Therefore, information about the color change is still important as an index of the loss of quality. Analyses performed on all products obtained by different processes from fresh or defrosted samples gave the results reported in Table S1 (Supplementary material), through which it is possible to observe the wide range covered by the data. L* is the index of sample lightness, ranges between about 33.3 and 41.3 denoting browning or bleaching in dependence of the adopted processing; a*, whose positive values indicate the sample redness, ranges between about 17.1 and 21.9, and b* positive values, an index of yellowness, range between about 6.9 and 11.5. Color intensity (C*_ab_), ranging between 18.7 and 24.3, and moreover color tonality, ranging between 21.6 and 28.2, show the existing differences among samples, all deriving by the same crop. It could be exemplified comparing the reflectance curves recorded after the same process from fresh (f) or defrosted (d) samples or from different heating or homogenization procedure (Figure 2).

All these samples show significant differences, more marked in the region between 550 and 700 nm and in the panel “D” are reported the profiles of samples with the strongest difference at all wavelengths. However, if we consider the behavior of the different series in general (type of homogenizer, fresh or defrosted starting material, dry heated by pasteurization or blanched with steam or microwaves treated), the differences do not allow us to define the influence of specific factors on color parameters. It also shows results relevant to the high variability of the sample highlighted in the high range of standard deviations recorded. Each sample denotes a different trend (also further confirmed by the HS–GC–MS analyses) which makes it difficult to point out general conclusions. The only evidence is the rising of lightness in blanched and/or defrosted samples, as well as after the microwave treatment. All blanched and defrosted samples show a red orange color nuance, with respect to the red pink color of the fresh pasteurized or the not heated samples.

Previous papers focus the anthocyanins stability to pH change, thermal treatments, light exposure, polyphenol oxidase influence, browning and co-pigmentation [24,25]. On the other hand, no data are available on the effects of different treatments on the same batch of strawberries. Our previous experiments demonstrated that a correlation could be evidenced between selected color parameters and anthocyanins ratio in blueberries and zeaxanthin content in goji [17,18]. In this case, such a correlation could not be as well depicted by the obtained data. If the differences between BUf (130 µg/g fw anthocyanins) and PUf (260 µg/g) could be explained by the different anthocyanins content, it is not possible to explain the similar trends of BUf and PUd (130 µg/g), with respect to BUd (40 µg/g). As, to our knowledge, no results are reported in the literature about this context, we could only hypothesize that the steam blanching, although impacting on the anthocyanin degradation, could preserve the sample against the enzymatic browning. On the contrary, while protecting the anthocyanin content, the pasteurization process induces a slight browning process. Finally, the thawing, although impacting more drastically on anthocyanins content, preserves the samples color on the whole.

In any case, a direct correlation between color and anthocyanin content could not be shown and a more complex flavonoid composition (high content of flavanols with respect to flavonols, perhaps a small carotenoid content) and counteracting browning and/or bleaching processes [26] need to be taken into consideration. For this reason, the color appearance allows us to discriminate among differently treated samples, only if correlated with all the other monitored parameters, as further shown by the PCA analysis.

### 3.4. HS-GC/MS Analysis

Ten components, listed in Table 1 and Table 2, were identified by HS–GC/MS of the different analyzed samples of Clery strawberries. In Table 1 the results of the analyses performed on fresh plant material are reported, comparing the two different homogenizations alone or after microwave or blanching treatments. Ethyl acetate and methyl butanoate were revealed in all samples, with the highest percentage of ethyl acetate (30.4%) in M sample, and the highest percentage of methyl butanoate (22.2%) in U. The most represented molecule in the only homogenized and microwave-treated samples (M, U, WM, WU) was methyl acetate, which accounted for about 35–40%. On the contrary, it was completely absent in the blanched samples (BM, BU), in which a high content of ethanol was found (60–70%). A low content of methyl hexanoate (6.9%) and of ethyl hexanoate (1.7%), in the sliced strawberries, has been found in the simply homogenized (U and M) samples.

BM and BU showed a similar chemical composition, with the only difference in the acetaldehyde and acetone content. Sliced strawberry, M and U samples displayed the same chemical qualitative profile, but the M sample also contained a very small amount of 2-hydroxy propanamide.

In Table 2, the results obtained by the analysis of all the pasteurized samples are reported, comprising two defrosted samples (PMd and PUd). After the blanching treatment after the pasteurization, only few components were found and a high quantity of ethanol was expressed. The high percentage of ethanol in blanched or pasteurized samples before the application of the homogenization process (BM, BU, PM, PU, PMd and PUd) could be justified by the heating induction of an accelerated fermentation process during the next grinding process [27]. Methyl acetate, ethyl butanoate, 2–hydroxy propanamide, methyl and ethyl hexanoate had completely disappeared after the thermal treatment.

All these samples are characterized for about the 90% by two only molecules which are represented by acetone and methyl butanoate (in MP and BUP), ethyl acetate and ethanol (in UP, PM, PMd, PUd), acetone and ethanol (in PU), acetone and ethyl acetate (in BMP), thus indicating a substantial modification and decrease of the aromatic character.

According to the literature, the more impacting molecules on strawberries’ flavors are recognized in two furfural derivatives, ethyl hexanoate, hexanal, ethyl methyl butanoate and methyl butanoate [28]. The high impact on the volatile components, besides the specific cultivar, is also due to cultural techniques, shading and harvesting dates [29]. Regarding the ester derivatives, the impact on fruity and floral character is generally recognized. Although less important for the aroma character, methyl acetate, which we found as the most represented in the sliced strawberries, was also indicated by Watson et al. [28] as the highest peak in Elsanta cultivar. Ethyl hexanoate was well represented and found in seven of the eight strawberry cultivars analyzed by Oz et al. [29]; whereas methyl butanoate and ethyl butanoate were found in half of the samples, methyl butanoate and ethyl acetate (only in trace) were found in only one case. Ethyl hexanoate was also reported by Kafkas et al. in strawberry wine [30].

The decrease of these ester compounds with respect to the sliced strawberry used as comparator seems to represent a good index to evaluate the treatment impact on the aroma value. These modifications are more evident in the representation reported in Figures S1 and S2 (Supplementary Materials).

### 3.5. HPLC–Analysis

The hydroalcoholic extracts of all the homogenized samples were then subjected to HPLC–DAD analysis. The chromatograms registered at 280 nm, 360 nm and 520 nm are reported in Figure S3 (Supplementary Materials).

Many different hydroxycinnamic, flavonoid and anthocyanin molecules have been determined in the polyphenolic component of Clery strawberry cultivar, according to the different data shown by the literature [14,29,31]. By these reports, over twenty different anthocyanins were identified in different strawberry cultivars. The presence of anthocyanins in strawberry cultivars is confirmed in literature data. In particular, Kelebek et al. [31] observed in strawberries six different anthocyanins which were identified as cyanidin–3–glucoside, cyanidin–3–rutinoside, pelargonidin–3–glucoside, pelargonidin–3–rutinoside, pelargonidin–3–malonyl-glucoside and pelargonidin–3–acetyl–glucoside. In our analyzed samples, we could observe a very simple profile (Figure S3, Panel C), with regard to the anthocyanin content. A slightly more complex profile was shown by the chromatogram at 280 nm (Figure S3, Panel A) that allows the identification of three hydroxycinnamic acids (namely ferulic, caffeic and *p*–coumaric acid) and the two flavan–3–ols (namely catechin and epicatechin). A more complex chromatogram registered at 360 nm (Figure S3, Panel B), in which only quercetin–3–D–galactoside was identified, but some peaks remained unresolved after direct comparison with pure standard or data on literature [31,32]. In Table 3, the data of the quantitative HPLC analyses on the fresh and the defrosted series are reported. Anthocyanins were quantified by a calibration curve, built on the pure reference standard of pelargonidin–3–glucoside, and were expressed as the sum of all the peaks areas monitored at 520 nm.

As shown in Table 3, the total amount of anthocyanins, expressed as µg of pelargonidin–3–glucoside/g fw, is much higher in the fresh, not frozen, samples than in defrosted ones (d). In particular, in the fresh series, the amounts of anthocyanins range between about 120 µg/g fw in BM and 260 µg/g fw in PU, whereas, in the defrosted series (d), it varies from about 30 µg/g fw in UP to about 160 µg/g fw in U.

Buendia et al. [33] reported that the total anthocyanin content of different strawberry cultivars ranged from 202 to 466 µg/g fw, whereas in a study conducted on the fruits of cv. Clery during ripening [32], the total anthocyanin content in full ripe fruits is about 430 µg/g fw. Gasperotti et al. [9] reported an anthocyanin content of about 360 µg/g fw.

By our results, it is clearly shown, both in the fresh and in the thawed series, that significant differences of anthocyanins amounts are associated to the different applied treatments. It could be observed that, on average, the amounts of anthocyanins in the pasteurized samples (PM, PU, MP and UP) are much higher than in samples subjected to blanching (BM, BU, BMP and BUP). More specifically, in the fresh series, the average amount of anthocyanins in the pasteurized samples is almost double (about 236 µg/g fw) than in the blanched samples (about 134 µg/g fw), in which a decrease in anthocyanin content of about 44% is observed.

In a previous work, it was reported the high impact of a steam blanching treatment, with respect to a pasteurization process, both performed similarly to what we did. Although they hypothesized a browning due to anthocyanins polymerization, we did not see such effect on color appearance. On the other hand, we think the direct exposition of the fruits to the penetrating power of steam could have a different effect with respect to the dry heating of fruits. This could act on polyphenol oxidase inactivation with prevention of enzymatic browning, nevertheless impacting on anthocyanin stability. It must be also taken in consideration that different anthocyanins were here represented and many other variables could influence their modification, such as vitamin C content, other polyphenolic content and so on [34].

On the contrary, in the d series, the average amount of anthocyanins in pasteurized samples was only slightly higher (77 vs. 53 µg/g fw) than in the blanched samples, showing that the freezing and thawing process provokes an evident anthocyanin content loss and a partially equilibrating effect. In fact, there is a loss of about 70% of the anthocyanin content in thawed samples compared to fresh treatments, with the exception of Ud and BMd where there is a loss of only 30%.

With regards to the procyanidins in the fresh samples, catechin ranged between about 100 (in UP) and about 250 µg/g fw (in U), whereas, it completely lacks in M and BM, as well as in the defrosted samples. Epicatechin, more represented, varies between 170 and 315 in the fresh series, whereas it decreases to 75 and 290 in the d series, with a loss of catechin content of about 63% in thawed samples. Therefore, higher values of flavan-3-ols were confirmed in the not thawed series, as for the anthocyanin content, and also if a different variability is shown between different couples of samples. Much lower are the contents of *p*–coumaric acid (5–60 µg/g fw) and caffeic acid (about 10–90 µg/g fw), whereas the ferulic acid derivatives are a little more represented (60–240 µg/g fw). In the flavonoid scaffold, monitored at 360 nm, a very small content of rutin was quantified (between 1.1 and 13.5 µg/g fw) in the fresh samples, but it was not detected in the d series.

The sum of the areas related to the other shown peaks, among which kaempferol, was expressed as quercetin–3–D–galactoside. These quantified flavonoids ranged between 2 and 28 µg/g fw in the fresh series and was equally represented in the d series (5–28 µg/g fw). All these results are, as general consideration, in agreement with other data published in literature which report ranges of 15–60 µg/g fw for flavanols, 15–30 µg/g fw for flavonols, 20 35 µg/g fw for cinnamic acids, 10–50 µg/g fw for phenolic acids and less than 10 µg/g fw for quercetin and kaempferol [31,32,33,35].

By the overview of the all obtained results, it could be possible to conclude that, if the thawing has caused an important decrease in the amounts of anthocyanins, it did not impact on the hydroxycinnamic content. Rather, the average content of caffeic, ferulic and *p*–coumaric acids in the d series seems to be perfectly preserved, if related to the content found after other treatments.

On the contrary, the freezing and defrosting cause a severe impact on catechin and rutin that results in secondary effects from a quantitative point of view and results as a relevant modification of qualitative profile. The amount of other flavonoid metabolites (e.g., epicatechin) are not significantly affected by processes applied [36].

**Figure 3:**
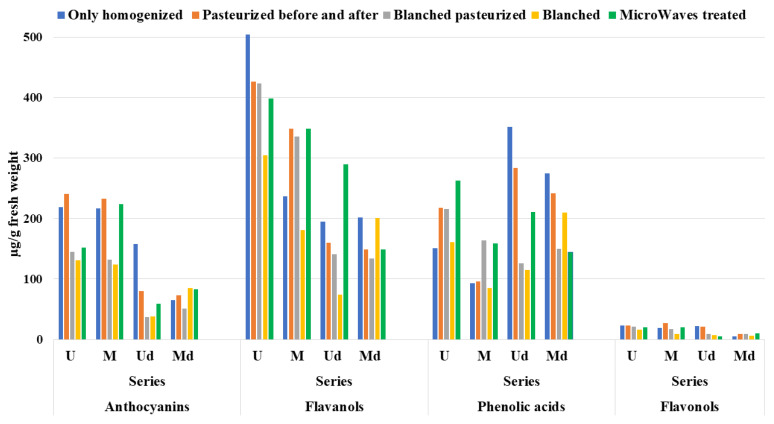
Effects of the different treatments applied to the strawberry cultivar Clery, U and M series, on the phytocomplex.

As far as concerns the homogenization process, a generally higher polyphenolic content can be observed in samples subjected to the Ultraturrax^®^ treatment, in both series (Figure 3).

In particular, it seems that the different type of homogenization (domestic mixer or Ultraturrax^®^) does not affect the anthocyanin and flavonol content as much as the content of flavanols and phenolic acids, which account for an average loss of 35% in series f treated with domestic mixer, much less marked in thawed samples (about 4.5%). The hydroxycinnamic and flavonoid contents are influenced by the different carried out thermal treatments, although to a less drastic extent if compared to anthocyanins, indicating that these last pigments are much more sensitive, particularly to the thawing.

The effect of thawing on anthocyanins degradation was previously reported and discussed by Holzwarth et al. [37], which also reported the different impact of thawing at different temperature and time conditions. Anthocyanins degradation between about 7% and 21% was shown, dependently on the different applied method. These results only partially agree with ours, by which a decrease of anthocyanins of 27% was shown in the sample only homogenized in Ultraturrax^®^ after thawing. Although frozen at -80 °C, avoiding the tissue destruction, and thawed at room temperature in less than an hour for their small size, much higher degradation was shown for all the other defrosted samples, confirming the importance of the adopted working process in the whole.

### 3.6. Total Phenolic Content and Total Flavonoid Content Evaluation

In recent years, the chemical and biological properties of phenolic compounds are one of the most attractive subjects in the scientific studies. In this sense, we investigated the total phenolic and flavonoid content of the strawberry samples. As can be seen in Table 4, the highest amounts of phenolics and flavonoids were determined in MP (19.28 mg GAE/g and 2.53 mg RE/g, respectively). The lowest levels of phenolics and flavonoids were noted in WU (11.34 mg GAE/g) and BUP (0.83 mg RE/g), respectively.

In Table 5, the results relative to the antioxidant and antiradical properties exerted by the treated samples are reported. They are more consistent with TPC and TFC values than HPLC data, thus highlighting that other non-detected components could participate to these properties. As a general trend, all the samples displayed a different profile on the basis of the treatment(s). Homogenization with Ultraturrax^®^ led to the lowest results in terms of metal chelating activity and in the phosphomolybdenum assay (Ud), especially if previously subjected to the microwave irradiation (WU). As regards their antioxidant ability, investigated by five in vitro methods, the microwave treatment and the Ultraturrax^®^ homogenization had detrimental effects. The latter was partially tolerated only if associated to pasteurization or blanching approaches. The different impact of freezing can be seen comparing U–Ud and M-Md samples; the Ultraturrax^®^ homogenization must be preferred with respect to the domestic mixer if the samples are defrozen. Homogenization-pasteurization should be privileged to pasteurization-homogenization. Collectively, the best results according to the different mechanisms of antioxidant potential were obtained with the MP and PM samples suggesting that processing conditions strongly impact on strawberry content and healthy potential.

### 3.7. Principal Component Analysis (PCA)

**Figure 4:**
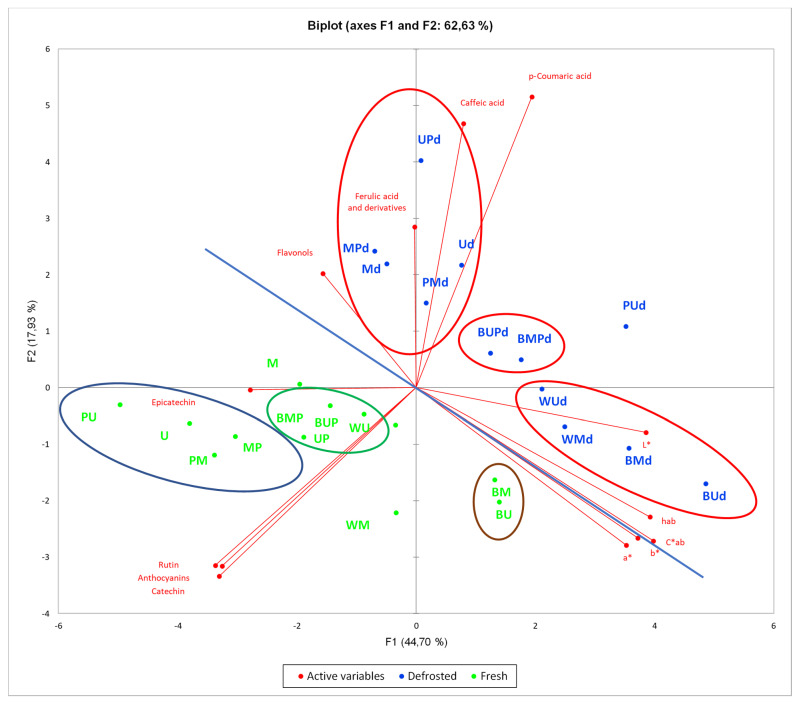
Principal component analysis (PCA) plots of the all strawberry Clery samples analyzed.

The PCA was carried out on all the data collected on the different series of samples in order to better find a correlation among the large variability of the reported data and samples. The values have been scaled, using XLSTAT 2020 software, with the unit variance scale. After scaling, all the parameters contributed in the same way on the variance and all variables acquired equal weight for the PCA. As shown in Figure 4, the *x*–axis represents the first PCA dimension (F1) covering the 44% of the total variance, whereas *y*–axis is the second PCA dimension (F2, 18% of the total variance). The red vectors indicate the investigated variables. The vector lengths are an index of the representativeness of the investigated PCA dimensions.

The most significant variables to discriminate between fresh and defrosted samples are represented by phenolic acids content, which is higher in the defrosted samples, whereas flavonoids are more represented in the fresh ones. The CIEL*a*b* parameters seem to contribute less. Considering the different analyzed variables (Figure 4), a clear separation between fresh (green) and defrosted (blue) samples could be revealed. Moreover, in both series, a separation into different groups could be underlined.

**Figure 5:**
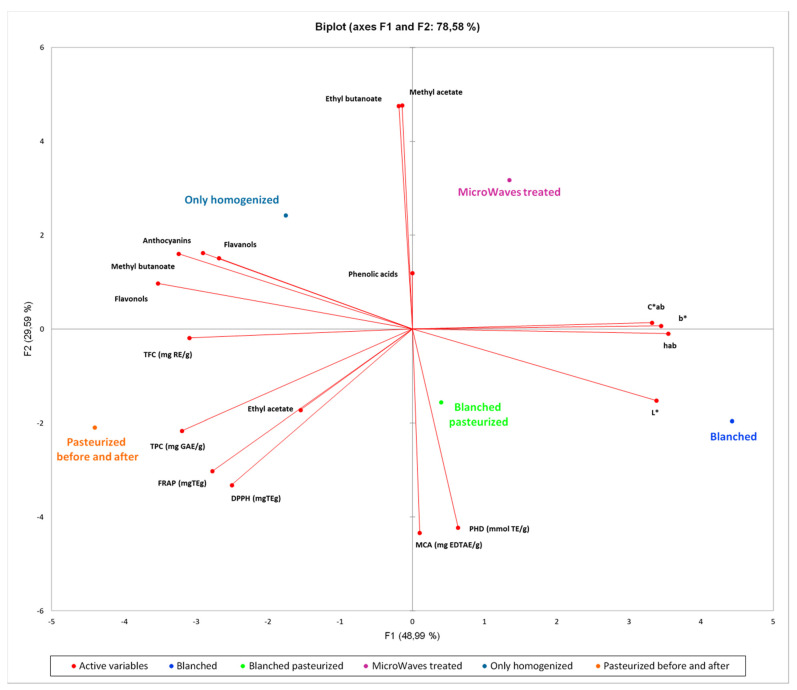
Principal component analysis (PCA) plots of the fresh analyzed samples evaluated as series.

This could be better explained by Figure S4 (in Supplementary Materials) and in Figure 5, in which the effect of different treatments is compared within the fresh sample series. The pasteurized samples represent the most interesting ones in terms of TPC, TFC, antioxidant and antiradical effects with respect to the corresponding homogenates samples. Both the blanching and microwave treatment induce a relevant impact in quality parameters, while the pasteurization performed immediately after blanching (green samples) seems to preserve from modification induced by the blanching process.

## 4. Conclusions

The healthy potential of the consumption of fruits and vegetables is strongly related to the industrial and domestic processing treatments aiming at improving the shelf life of seasonal products and limiting the loss of bioactive components.

Overall, in treated strawberry samples, the best flow chart in terms of bioactives preservation is represented by the mild pasteurization procedure, performed before or after the homogenization process. In this regard, probably related to the swollen tissue that represents the edible part of the fruit, the use of domestic mixer results in an efficient homogenization system, supporting the possibility to activate productive chain with economic and friendly-use instruments. The pasteurization, both performed before or after homogenization and performed after blanching plus homogenization, determines a darker reddish color product, which could account for a very slight browning. We can speculate that this color change is related to enzyme mediated oxidative processes, for example, polyphenol oxidase, that was only partially inactivated by the blanching treatments or by the microwave treatment, in any case performed before the homogenization process. In conclusion, not pasteurized samples are characterized by a red brilliant, perhaps more pleasant color, which significantly affects the economic value of these products on the market and better preservation of some aroma compounds, whereas the pasteurized samples account for the best nutraceutical properties, maintaining a higher amount of polyphenolic compounds and their antioxidant potential. The PCA analysis of the collected data further corroborated the more suitable processing for this natural matrix.

## Figures and Tables

**Table 1:** Fresh strawberry HS-GC peak area (%).

Components ^1^	LRI ^2^	LRI ^3^	Sliced	U	M	WU	WM	BM	BU
Acetaldehyde	650	655	3.8	12.9	4.4	5.7	8.5	2.8	-
Methyl acetate	826	828	40.0	34.6	36.7	38.7	38.8	-	-
Acetone	838	842	1.9	9.9	4.4	6.9	4.9	-	11.5
Ethyl acetate	880	885	19.8	5.8	30.4	27.1	25.4	23.9	20.4
Methyl butanoate	986	985	17.7	22.2	9.6	7.9	9.2	2.4	9.1
Ethanol			nd	nd	nd	nd	nd	70.9	59.0
Ethyl butanoate	1040	1036	8.1	8.9	7.9	8.3	8.8	nd	nd
2-hydroxy propanamide	1114	*	-	-	2.6	-	4.4	-	-
Methyl hexanoate	1182	1187	6.9	2.1	3.5	-	-	-	-
Ethyl hexanoate	1234	1238	1.7	3.4	0.4	-	-	-	-
Sum			99.9	99.8	99.9	94.6	100.0	100.0	100.0

^1^ Elution order on polar column; ^2^ linear retention indices (LRI) measured on polar column; ^3^ linear retention indices from literature; * LRI^lit^ not available; traces < 0.1%; sliced: untreated strawberry.

**Table 2:** HS-GC peak area (%) of fresh and defrosted (d) strawberries to which was applied a pasteurization process before or after homogenization or after blanching and homogenization.

Components ^1^	LRI ^2^	LRI ^3^	MP	UP	PM	PU	BMP	BUP	PMd	PUd
Acetaldehyde	650	655	-	1.9	1.3	-	-	-	6.0	2.3
Acetone	838	842	57.4	8.6	0.4	26.9	53.1	76.4	-	0.8
Ethyl acetate	880	885	-	61.4	61.4	2.9	46.9	-	33.9	16.2
Ethanol	940	938	-	25.3	36.9	70.2	-	-	60.1	80.7
Methyl butanoate	986	985	42.6	2.8	-	-	-	23.6	-	-
Sum			100.0	100.0	100.0	100.0	100.0	100.0	100.0	100.0

^1^ Elution order on polar column; ^2^ linear retention indices measured on polar column; ^3^ linear retention indices from literature.

**Table 3:** HPLC-DAD quantitative analysis.

	M	U	BM	BU	BMP	BUP	PM	PU	MP	UP	WM	WU
**Catechin**	-	253.7	-	108.9	98.1	135.1	177.5	210.8	165.3	96.6	153.4	178.4
**Epicatechin**	236.4	251.4	181.2	195.6	237.6	288.8	184.4	316.4	168.5	229.5	195.9	220.8
**Caffeic acid**	11.8	10.8	11.7	12.2	10.0	18.2	8.4	18.5	7.2	14.4	17.8	48.9
***p*–Coumaric acid**	5.6	5.9	-	7.6	13.1	17.1	4.8	15.1	11.2	16.5	8.2	13.4
Ferulic acid and derivatives *	75.8	134.4	73.7	141.6	140.6	180.7	56.2	197.3	103.9	173.2	133.1	200.6
**Rutin**	-	4.8	1.1	3.4	6.1	5.4	6.5	13.5	9.8	10.8	7.1	7.1
Flavonols **	19.3	18.2	1.6	12.2	10.8	15.4	8.4	8.5	28.4	14.1	12.8	13.2
Anthocyanins ***	216.6	219.1	123.5	131.4	131.7	144.6	237.9	258.5	227.2	222.6	223.5	151.7
**Sum**	565.5	898.3	398.8	612.9	648.0	805.3	684.1	1038.6	721.5	777.7	751.8	834.1
	**Md**	**Ud**	**BMd**	**BUd**	**BMPd**	**BUPd**	**PMd**	**PUd**	**MPd**	**UPd**	**WMd**	**WUd**
**Epicatechin**	202.3	194.8	200.7	73.9	133.5	141.4	157.8	126.5	222.6	192.7	148.6	290.2
**Caffeic acid**	38.6	77.2	20.0	5.8	31.7	5.7	31.0	88.7	47.9	54.5	nd	nd
***p*-Coumaric acid**	20.4	31.7	22.8	26.2	25.3	29.9	23.2	34.3	19.7	62.2	21.3	31.8
Ferulic acid and derivatives *	216.2	242.5	167.2	83.2	92.7	90.4	134.4	207.8	227.9	120.9	122.9	178.8
Flavonols **	5.3	21.7	5.9	6.9	8.6	9.0	8.3	13.4	10.5	28.1	10.3	5.4
Anthocyanins ***	64.9	158.3	85.2	37.9	50.6	36.8	69.4	129.7	76.8	30.4	82.6	58.8
**Sum**	547.7	726.2	501.8	233.8	342.4	313.2	424.1	600.4	605.4	488.8	385.7	565.0

Results are expressed in µg/g of fresh weight. The RSD value, evaluated on triplicates, was < 5%. Catechin and Rutin, although detected, were not revealed in the defrosted samples. * Expressed as ferulic acid. ** Expressed as quercetin-3-D-galactoside. *** Expressed as pelargonidin–3–glucoside.

**Table 4:** Total phenolic and flavonoid contents in the strawberry samples ^*^.

Samples	TPC (mg GAE/g)	TFC (mg RE/g)
BUP	14.63 ± 0.22 ^d^	0.83 ± 0.05 ^f^
BMP	12.66 ± 0.13 ^f^	1.14 ± 0.04 ^d^
U	12.99 ± 0.10 ^f^	0.91 ± 0.04 ^f^
M	13.69 ± 0.12 ^e^	1.54 ± 0.06 ^bc^
UP	14.39 ± 0.15 ^d^	1.17 ± 0.03 ^d^
MP	19.28 ± 0.06 ^a^	2.53 ± 0.03 ^a^
PU	16.38 ± 0.13 ^c^	1.06 ± 0.07 ^de^
PM	17.27 ± 0.21 ^b^	1.59 ± 0.05 ^b^
WU	11.34 ± 0.15 ^h^	1.19 ± 0.03 ^d^
WM	13.83 ± 0.11 ^e^	1.19 ± 0.05 ^d^
BU	12.84 ± 0.03 ^f^	0.96 ± 0.05 ^ef^
BM	11.97 ± 0.10 ^g^	1.08 ± 0.05 ^de^
Ud	12.16 ± 0.08 ^g^	1.16 ± 0.06 ^d^
Md	13.67 ± 0.15 ^e^	1.40 ± 0.05 ^c^

* Values are reported as mean ± S.D. TPC: total phenolic content; TFC: total flavonoid content; GAE: gallic acid equivalent; RE: rutin equivalent; different letters indicate significant differences in the samples (*p* < 0.05).

**Table 5:** Antioxidant properties of the strawberry samples *.

Samples	DPPH (mg TE/g)	ABTS (mg TE/g)	CUPRAC (mg TE/g)	FRAP (mg/TE)	MCA (mg EDTAE/g)	PHD (mmol TE/g)
BUP	27.92±0.53 ^b^	38.19 ± 0.19 ^c^	56.45 ± 0.33 ^c^	38.81 ± 0.57 ^c^	7.73 ± 0.09 ^a^	1.22 ± 0.13 ^abc^
BMP	23.65 ± 0.32 ^c^	33.50 ± 0.13 ^e^	49.55 ± 0.24 ^ef^	33.58 ± 0.19 ^ef^	7.06 ± 0.06 ^ab^	1.09 ± 0.03 ^bc^
U	21.73 ± 0.24 ^de^	31.47 ± 0.41 ^f^	48.57 ± 0.40 ^f^	32.52 ± 0.38 ^f^	4.57 ± 0.78 ^ef^	1.06 ± 0.07 ^bc^
M	21.11 ± 0.35 ^e^	31.73 ± 0.53 ^f^	48.85 ± 0.18 ^ef^	33.22 ± 0.29 ^ef^	3.89 ± 0.32 ^f^	1.13 ± 0.14 ^abc^
UP	27.17 ± 0.50 ^b^	36.35 ± 0.93 ^d^	54.92 ± 0.31 ^c^	37.47 ± 0.61 ^d^	3.65 ± 0.20 ^f^	1.10 ± 0.04 ^abc^
MP	33.20 ± 0.96 ^a^	45.41 ± 0.35 ^a^	72.26 ± 0.42 ^a^	48.11 ± 0.50 ^a^	6.87 ± 0.21 ^abc^	1.27 ± 0.09 ^ab^
PU	26.79 ± 0.26 ^b^	38.19 ± 0.28 ^c^	61.02 ± 0.67 ^b^	39.15 ± 0.16 ^c^	6.09 ± 0.12 ^bcd^	1.13 ± 0.03 ^abc^
PM	27.29 ± 0.61 ^b^	40.98 ± 0.85 ^b^	61.76 ± 1.05 ^b^	42.15 ± 0.34 ^b^	6.98 ± 0.48 ^abc^	1.17 ± 0.08 ^abc^
WU	18.47 ± 0.51 ^f^	25.52 ± 0.66 ^i^	35.30 ± 0.18 ^g^	26.90 ± 0.44 ^h^	1.91 ± 0.14 ^g^	0.95 ± 0.07 ^c^
WM	21.70 ± 0.50 ^de^	32.52 ± 0.09 ^ef^	50.38 ± 1.31 ^e^	36.31 ± 0.17 ^d^	2.18 ± 0.44 ^g^	1.03 ± 0.04 ^bc^
BU	21.64 ± 0.09 ^de^	28.49 ± 0.45 ^gh^	52.79 ± 0.72 ^d^	34.41 ± 0.50 ^e^	5.98 ± 0.25 ^cd^	1.23 ± 0.11 ^ab^
BM	20.95 ± 0.50 ^e^	29.87 ± 0.17 ^g^	47.98 ± 0.61 ^f^	30.59 ± 0.47 ^g^	6.92 ± 0.30 ^abc^	1.26 ± 0.16 ^ab^
Ud	21.47 ± 0.24 ^de^	28.16 ± 0.81 ^h^	48.33 ± 0.25 ^f^	32.40 ± 0.24 ^f^	1.64 ± 0.24 ^g^	1.38 ± 0.12 ^a^
Md	22.73 ± 0.09 ^cd^	32.25 ± 0.32 ^ef^	52.42 ± 0.39 ^d^	34.26 ± 0.55 ^e^	5.57 ± 0.57 ^de^	1.30 ± 0.07 ^ab^

* Values are reported as mean ± S.D. TE: trolox equivalent; EDTAE: EDTA equivalent; MCA: metal chelating assay; PHD: phosphomolybdenum assay; different letters indicate significant differences in the samples (*p* < 0.05).

## Supplementary Materials

The following are available online at https://www.mdpi.com/2076-3921/9/7/632/s1, Figures S1 and S2: Quantified (%) single compounds of analyzed strawberry samples. Figure S3. Chromatograms of the strawberry hydroalcoholic extracts. Figure S4. Principal component analysis (PCA) plots of the fresh analyzed samples. Table S1: Colorimetric CIEL*a*b* parameters of strawberries homogenates.
